# ASB2 is a direct target of FLI1 that sustains NF-κB pathway activation in germinal center-derived diffuse large B-cell lymphoma

**DOI:** 10.1186/s13046-021-02159-3

**Published:** 2021-11-11

**Authors:** Giulio Sartori, Sara Napoli, Luciano Cascione, Elaine Yee Lin Chung, Valdemar Priebe, Alberto Jesus Arribas, Afua Adjeiwaa Mensah, Michela Dall’Angelo, Chiara Falzarano, Laura Barnabei, Mattia Forcato, Andrea Rinaldi, Silvio Bicciato, Margot Thome, Francesco Bertoni

**Affiliations:** 1 Institute of Oncology Research, Faculty of Biomedical Sciences, USI, via Francesco Chiesa 5, 6500 Bellinzona, Switzerland; 2 Swiss Institute of Bioinformatics, Lausanne, Switzerland; 3 Department of Computer Science, University of Verona, Verona, Italy; 4 Department of Life Sciences, University of Modena and Reggio Emilia, Modena, Italy; 5 Department of Biochemistry, University of Lausanne, Epalinges, Switzerland; 6 Oncology Institute of Southern Switzerland (IOSI), Bellinzona, Switzerland

**Keywords:** 11q24.3 gain, Diffuse large B-cell lymphoma (DLBCL), Transcription factor FLI1, NFKB pathway, ASB2

## Abstract

**Background:**

Diffuse large B-cell lymphoma (DLBCL) comprises at least two main biologically distinct entities: germinal center B-cell (GCB) and activated B-cell (ABC) subtype. Albeit sharing common lesions, GCB and ABC DLBCL present subtype-specific oncogenic pathway perturbations. ABC DLBCL is typically characterized by a constitutively active NF-kB. However, the latter is seen in also 30% of GCB DLBCL. Another recurrent lesion in DLBCL is an 11q24.3 gain, associated with the overexpression of two ETS transcription factors, ETS1 and FLI1. Here, we showed that FLI1 is more expressed in GCB than ABC DLBCL and we characterized its transcriptional network.

**Methods:**

Gene expression data were obtained from public datasets GSE98588, phs001444.v2.p1, GSE95013 and GSE10846. ChIP-Seq for FLI1 paired with transcriptome analysis (RNA-Seq) after FLI1 silencing (siRNAs) was performed. Sequencing was carried out using the NextSeq 500 (Illumina). Detection of peaks was done using HOMER (v2.6); differential expressed genes were identified using moderated t-test (limma R-package) and functionally annotated with g:Profiler. ChIP-Seq and RNA-Seq data from GCB DLBCL cell lines after FLI1 downregulation were integrated to identify putative direct targets of FLI1.

**Results:**

Analysis of clinical DLBCL specimens showed that FLI1 gene was more frequently expressed at higher levels in GCB than in ABC DLBCL and its  protein levels were higher in GCB than in ABC DLBCL cell lines**.** Genes negatively regulated by FLI1 included tumor suppressor genes involved in negative regulation of cell cycle and hypoxia. Among positively regulated targets of FLI1, we found genes annotated for immune response, MYC targets, NF-κB and BCR signaling and NOTCH pathway genes. Of note, direct targets of FLI1 overlapped with genes regulated by ETS1, the other transcription factor gained at the 11q24.3 locus in DLBCL, suggesting a functional convergence within the ETS family. Positive targets of FLI1 included the NF-κB-associated *ASB2*, a putative essential gene for DLBCL cell survival. *ASB2* gene downregulation was toxic in GCB DLBCL cell lines and induced NF-κB inhibition via downregulation of RelB and increased IκBα. Additionally, downregulation of *FLI1*, but not *ASB2*, caused reduction of NF-κB1 and RelA protein levels.

**Conclusions:**

We conclude that FLI1 directly regulates a network of biologically crucial genes and processes in GCB DLBCL. FLI1 regulates both the classical NF-κB pathway at the transcriptional level, and the alternative NF-κB pathway, via ASB2. FLI1 and ASB2 inhibition represents a potential novel therapeutic approach for GCB DLBCL.

**Supplementary Information:**

The online version contains supplementary material available at 10.1186/s13046-021-02159-3.

## Background

Diffuse large B-cell lymphoma (DLBCL) is the most common lymphoma type, and it comprises at least two main biologically distinct entities that are referred to as germinal center B-cell (GCB) and activated B-cell (ABC) subtype [1–3]. GCB and ABC DLBCL both share common genetic lesions and present subtype-specific alterations. ABC DLBCL is typically characterized by specific oncogenic pathway perturbations leading to a constitutively active NF-κB. However, the latter is not specific for the ABC subtype, and classical and alternative NF-κB pathways can be activated in both subtypes [4]. Indeed, over 60% of ABC-DLBCL and 30% of GCB DLBCL present nuclear localization of NFKB1/p50, compatible with an active classical NF-κB pathway, and/or of NFKB2/p52, as read out of an active alternative pathway [4]. The distinction between GCB and ABC DLBCL has been improved by the identification of series of genetically defined subclusters including the largely overlapping MCD and C5, exclusively comprising ABC DLBCL and the C3 and EZB enriched in GCB DLBCL [2, 3, 5, 6]. Another recurrent lesion in DLBCL is an 11q24.3 gain, observed in up to one quarter of cases resulting in deregulation of ETS1 (ETS Proto-Oncogene 1) and FLI1 (Friend Leukemia Insertion 1), two ETS family transcription factors that contribute to DLBCL pathogenesis [7, 8]. ETS1 is more expressed in ABC than in GCB DLBCL and it regulates genes involved in B-cell signaling, differentiation and cell cycle [8, 9]. Less is known regarding the role of FLI1 in DLBCL. Our initial study reported that FLI1 modulated genes and pathways only partially overlapping with ETS1 [8]. The *FLI1* gene is an oncogene rearranged in 95% of Ewing sarcoma, a pediatric tumor of neuroectodermal origin [10–12]. In this type of sarcoma, the translocation t(11;22)(q24;q12) occurs between the central exons of FLI1 and the central exons of Ewing sarcoma breakpoint region1 (EWSR1) on chromosome 22, creating a fusion protein with dual transcriptional activator and repressor function [13–16]. In normal tissues, FLI1 is transiently expressed during embryogenesis, and in adults it is highly expressed in hematopoietic tissue and endothelial cells with lower levels detected in lung, heart and ovaries [17]. FLI1 is involved in angiogenesis, differentiation of megakaryocytes, cell cycle promotion and inhibition of apoptosis [15]. Overexpression of FLI1 in transgenic mice results in the development of a lupus-like disease, including hypergammaglobulinemia, splenomegaly, B-cell peripheral lymphocytosis, progressive immune complex-mediated renal disease and ultimately premature death from renal failure [18]. In contrast, reduced expression of FLI1 in MRL/lpr mice, a murine model of lupus, significantly increases survival and decreases renal disease compared with wild type counterparts [19]. Mice with reduced levels of FLI1 have reduced Igα expression and this reduction may contribute to decreased BCR signaling, fewer follicular B cells and an increased number of marginal zone B cells [20]. Immune responses and in vitro class switch recombination are altered in FLI1-deficient mice [20]. Taken together, these studies suggest that FLI1 plays an important role in immune cells including the B-cell compartment. Here, we defined the transcriptional network regulated by FLI1 in GCB DLBCL, which expresses higher levels of FLI1 than ABC DLBCL.

## Methods

### RNA expression datasets and cell lines

Publicly available expression datasets of DLBCL clinical specimens obtained with RNA-Seq or Affymetrix Genechip U133 plus 2.0 were used: GSE98588, phs001444.v2.p1, GSE95013 and GSE10846 [5, 6, 21, 22]. The CEL raw data files were imported and preprocessed by log2 transformation with normalization using Bioconductor packages in R Studio: voom/limma [23, 24] and edgeR [25]. FLI1 mRNA expression was dichotomized into high and low values using the median as a cut-off for further analyses. The GSE10846 dataset consisted of two separate series of specimens, which were batch corrected.

Cell lines were cultured under standard conditions at 37 °C in a humidified atmosphere, with 5% CO_2_. Twelve GCB cell lines (KARPAS-422, SU-DHL-4, SU-DHL-6, FARAGE, Pfeiffer, DoHH2, WSU-DLCL2, Toledo, OCI-Ly19, OCI-Ly8, OCI-Ly1, VAL) and eight ABC cell lines (HBL1, U2932, TMD8, SU-DHL-2, OCI-Ly3, OCI-Ly10, RCK8, RI-1) were obtained and maintained as previously described [26]. Cell lines identity was validated by STR DNA fingerprinting [26].

### Gene silencing

For transient knockdown we used the Amaxa 4D Nucleofector system (Lonza) to introduce three FLI1 siRNAs (J-003892-05, J-003892-06 and J-003892-08) or four ASB2 siRNAs from ON-TARGET SMARTpool siRNA (L-009575-00) or a non-targeting siRNA as control (Dharmacon GE Healthcare, now Horizon Discovery Ltd.). Protocols were followed according to the SG Cell Line 4D-Nucleofector X Kit L (Lonza). In brief, 2 × 10^6^ cells were prepared and resuspended in 100 μL SG solution with 500 nM siRNA or corresponding amounts of BLOCK-iT (Invitrogen) as a control for nucleofection efficiency. Efficiency was confirmed 48 h after nucleofection by flow cytometry and cells were harvested for protein lysates, RNA extraction and MTT assay as previously described [26].

### RNA extraction, PCR amplification and quantitative real-time PCR

RNA was isolated using Trizol (Invitrogen - Thermofisher Waltham MA, USA) and then DNAse-treated using RNase-free DNase Kit (Qiagen, Germantown, MD, USA). Total RNA extracts were reverse-transcribed using the SuperScript III First-strand Synthesis SuperMix System kit (Invitrogen) to generate cDNA. In brief, 800 ng of total RNA were mixed with 10 μL of 2x RT Reaction Mix and 2 μL RT Enzyme Mix and made up to a final volume of 20 μL with DEPC water (Invitrogen). Quantitative Real-Time (qRT)-PCR amplification was performed using the KAPA SYBR FAST qPCR Master Mix (2x) ABI Prism on a StepOnePlus Real-Time PCR system (Applied Biosystems). All primers were designed using the web-based program Primer3Plus (http://www.bioinformatics.nl/cgi-bin/primer3plus/primer3plus.cgi) in combination with PrimerBlast for validation of target specificity (https://www.ncbi.nlm.nih.gov/tools/primer-blast/). The thermal cycler was programmed as follows: Enzyme activation at 95 °C for 3′ followed by 40 cycles of denaturation (95 °C for 3 s) and annealing (60 °C for 30s) and finally, dissociation curve analysis. Primer efficiency was determined using linear modelling for the amplification curves with the LinReg software version 2015.4 [27]. Relative quantification was calculated using the Pfaffl method [28]. Primers targeting *FLI1*, *ASB2* and *GAPDH* are listed in Table S1.

### Immunoblotting

Cells were harvested and lysed by either boiling samples in 2x Laemmli sample buffer (BioRad) supplemented with β-mercaptoethanol (Merck) for 10′ or according to the manufacturer’s protocol using M-PER buffer (Thermo Fisher Scientific). Lysates (30–50 μg) were resolved according to molecular weight by electrophoresis using Mini-PROTEAN TGX Precast gels 4–20% gradient (BioRad). After electrophoresis proteins were blotted onto nitrocellulose membrane (BioRad) by electric transfer and the membranes were blocked in TBST (20 mM Tris-HCl [pH 7.5], 150 mM NaCl, 0.1% Tween 20) with 5% nonfat dry milk (BioRad) for 1 h at room temperature (RT). The following primary antibodies were used in TBST 5% BSA buffer: rabbit polyclonal α-FLI1 (ab-15,289, Abcam), rabbit polyclonal α-ASB2 (PA5–29476, Thermo Fisher Scientific), mouse monoclonal α-IkB-alpha (6A920) (NB100–56507, Novus), rabbit monoclonal α-NF-Kappa-B1 p105/p50 (D4P4D) (13,586, CST), rabbit monoclonal α-NF-Kappa-B2 p100/p52 (4882, CST), rabbit monoclonal NF-Kappa-B p65 (D14E12) (8242, CST), rabbit monoclonal α-RelB (C1E4) (4922,CST). Mouse monoclonal α-GAPDH (FF26A/F9, CNIO) was used in TBST with 5% nonfat dry milk. The secondary antibodies used were: ECL α-mouse IgG horseradish peroxidase-linked species-specific whole antibody and ECL α-Rabbit IgG horseradish peroxidase-linked species-specific whole antibody (GE Healthcare). Membranes were treated with Westar ηC 2.0 chemiluminescent substrate (Cyanagen) and signals were detected using digital imaging with Fusion Solo (Vilber Lourmat).

### Transcriptome analysis

Initial RNA quality control was performed on the Agilent BioAnalyzer (Agilent Technologies, California, USA) using the RNA 6000 Nano kit (Agilent Technologies) and concentration was determined with the Invitrogen Qubit (Thermo Fisher Scientific) using RNA BR reagents (Thermo Fisher Scientific). Total RNA samples were prepared for RNA-Seq with the NEBNext rRNA Depletion kit, the NEBNext Ultra Directional RNA Library Prep Kit for Illumina and NEBNext Multiplex Oligos for Illumina (New England BioLabs Inc.). Sequencing was performed using a NextSeq 500 with the NextSeq 500/550 High Output Kit v2 (150 cycles PE; Illumina). All data are available at the National Center for Biotechnology Information (NCBI) Gene Expression Omnibus (GEO) (http://www.ncbi.nlm.nih.gov/geo) database (GSE157191).

### Chromatin Immunoprecipitation (ChIP)

Chromatin was sheared with the M220 Focused ultrasonicator for Adaptive Focused Acoustics (AFA) technology (Covaris) using the milliTUBE 1 mL AFA fiber. The manufacturer’s protocol for the truCHIP Chromatin Shearing Kit was followed. 25 × 10^6 cells were washed in cold PBS and resuspended in Fixing Buffer A with 1% formaldehyde and mixed for 2′. After crosslinking the quenching buffer was added. Lysis of samples proceeded in accordance with the manufacturer’s protocol. The cell lysate suspension with chromatin was transferred into the milliTUBE and sonicated with the program set at 10% duty cycles with 200 cycles per burst for 12′. The quality of chromatin shearing was determined using the High Sensitivity DNA Analysis Kit (Agilent Technologies) and the 2100 BioAnalyzer (Agilent Technologies). ChIP was performed using 50 μL chromatin solutions (corresponding to 5 × 10^5 cells) diluted in ChIP dilution buffer (0.01% (w/v) SDS), 1.1% (v/v) Triton-X 100, 1.2 mM EDTA, 16.7 mM Tris–HCl, 167 mM NaCl [pH 8.1]) with 1x HALT Proteinase inhibitor cocktail (Thermo Scientific). Rabbit polyclonal α-FLI1, 5 μg (ab-15,289, Abcam) was added to the diluted chromatin samples. Antibody/protein/DNA complexes were captured with protein G magnetic beads at 4 °C (Millipore). Magnetic beads were washed using a magnetic rack and increasing stringencies of salt buffers in the following order: Low Salt washing buffer, 0.1% (w/v) SDS, 1%(v/v) Triton-X 100, 2 mM EDTA, 20 mM Tris–HCl [pH 8.1], 150 mM NaCl; High Salt washing buffer, (0.1% (w/v) SDS, Triton-X 100 1% (v/v), 2 mM EDTA [pH 8.0], 20 mM Tris–HCl [pH 8.1], 500 mM NaCl); LiCl buffer, 0.25 M LiCl, 1% (w/v) IGEPAL-CA 630, 1% (v/v) deoxycholic acid, 1 mM EDTA, 10 mM Tris–HCl [pH 8.1];Tris–EDTA buffer, (10 mM Tris–HCl, 1 mM EDTA, [pH 8.1]).The Tris EDTA buffer wash was repeated twice. Immunoprecipitated complexes were eluted from the dynabeads using elution buffer (SDS 1% (w/v), 0.1 M NaHCO3) with RNAse A added and incubated at 37 °C for 30′ on a thermomixer (1200 rpm). This was followed by reversal of cross-links performed by adding 5 M NaCl together with 0.5 M EDTA, Tris-HCL and Proteinase K for 2 h at 62 °C on a thermomixer (1200 rpm). Lastly DNA was purified using the QIAquick PCR purification kit (Qiagen). For validation, qRT-PCR was performed using KAPA SYBR FAST qPCR Master Mix (2x) ABI Prism. Primers targeting *ASB2, RASGRP1, AATF, DDX21* and *GAPDH* are listed in Table S1. For ChIP-Seq at least 5 parallel IPs were performed, and the eluted DNA was pulled and re-concentrated in 5 μL. All data will be available at the National Center for Biotechnology Information (NCBI) Gene Expression Omnibus (GEO) (http://www.ncbi.nlm.nih.gov/geo) database (GSE157191).

### Data mining

All bioinformatic processing was performed using R/Bioconductor software packages in RStudio. RNA-Seq raw reads were quality assessed using fastqc [29]. For each sample the distribution of unique, multi- and unmapped reads was checked for a high proportion of unmapped or multi mapped reads. Reads obtained from RNA sequencing were mapped against the human hg38 genome build using the Genecode version 22 annotation [30]. Alignment was done with STAR (v2.4.0 h) [31] and counting of reads overlapping gene features with HTSeq-Count [32]. Transcripts with a count-per-million greater than one in at least three samples underwent differential gene expression analysis was performed using the voom/limma [24] R package. Functional annotation was done with *g:Profiler* using gene sets from the Molecular Signatures Database (MSigDB v5.1) [33] (Hallmark, c2.all, c5.bp, c6), SignatureDB [34] and gene sets obtained from different publications as reported. *Standard settings were used for g:Profiler* data mining [35]*. Signatures with absolute log fold change > 0.1* and *adj.P < 0.05* were considered as biologically relevant.

For ChIP-Seq analysis, raw sequencing was mapped onto the Genome Reference Consortium Human Build 37 (GRCh37) using bowtie2. Reads filtering was done using SAMtools to keep reads that map only once, with a quality score of 10 or more, and to remove duplicates. We first performed an exploratory analysis with IGV genome browser to assess the quality of the ChIP and detect issues and abnormalities. Peaks were then called using HOMER and selected to control the false discovery rate (FDR) at 0.001. To biologically interpret the results of ChIP-Seq experiments, we looked at genes and other annotated elements that are located in proximity to the identified enriched regions (peak annotations) using HOMER, PeakAnalyzer and BedTools (version 2.17). Promoter regions were defined within 3 kb from the closest TSS.

Target peaks located further than 3 kb from the closest TSS were annotated using the enhancer-promoter interactions map of Mifsud et al. [36], derived from a Capture HiC (C-HiC) experiment from GM12878 cells, a human Epstein-Barr virus (EBV)-transformed lymphoblastoid cell line. Active enhancers overlapping with target peaks were assigned to the corresponding interacting promoter region.

Pearson correlation was used to identify those genes significantly (positively and negatively, *p* < 0.01) correlated with expression levels of FLI1 in DLBCL clinical specimens (GSE10846). Overlapping between lists was done using the VENNY on-line tool [37].

### Immunofluorescence staining and analysis

Cells were stimulated at 37 °C for 15 min with 10 μg/mL of goat F(ab′)2 anti–human IgM (Southern Biotech). Cells were coated on a poly-L-lysine matrix then fixed 20′ with PFA 4% at RT. Cells were permeabilized with PBS + 0.1% Triton X-100 10′ at RT. To block unspecific staining, samples were treated for 1 h with PBS + 5% BSA at RT before staining. The following primary antibody was used in PBS 5% BSA buffer: rabbit monoclonal α-NF-Kappa-B1 p105/p50 (D4P4D) (13,586, CST). Samples were incubated overnight at 4 °C. For immunofluorescence, the following secondary antibody was used: goat anti-rabbit IgG labelled with Alexa 568 (Thermo Fisher Scientific) 1 h at RT in the dark. Slides were counterstained after three washes of PBS with 0.3 μg/mL 4,6-diamidino-2-phenylindole (Sigma-Aldrich). Images including Z-stacks were acquired on a Leica SP5 with an objective with × 63 magnification. Nuclear localization of NF-κB1/p50 was quantified by ImageJ software.

## Results

### FLI1 is more highly expressed in GCB than ABC DLBCL

**Fig. 1:**
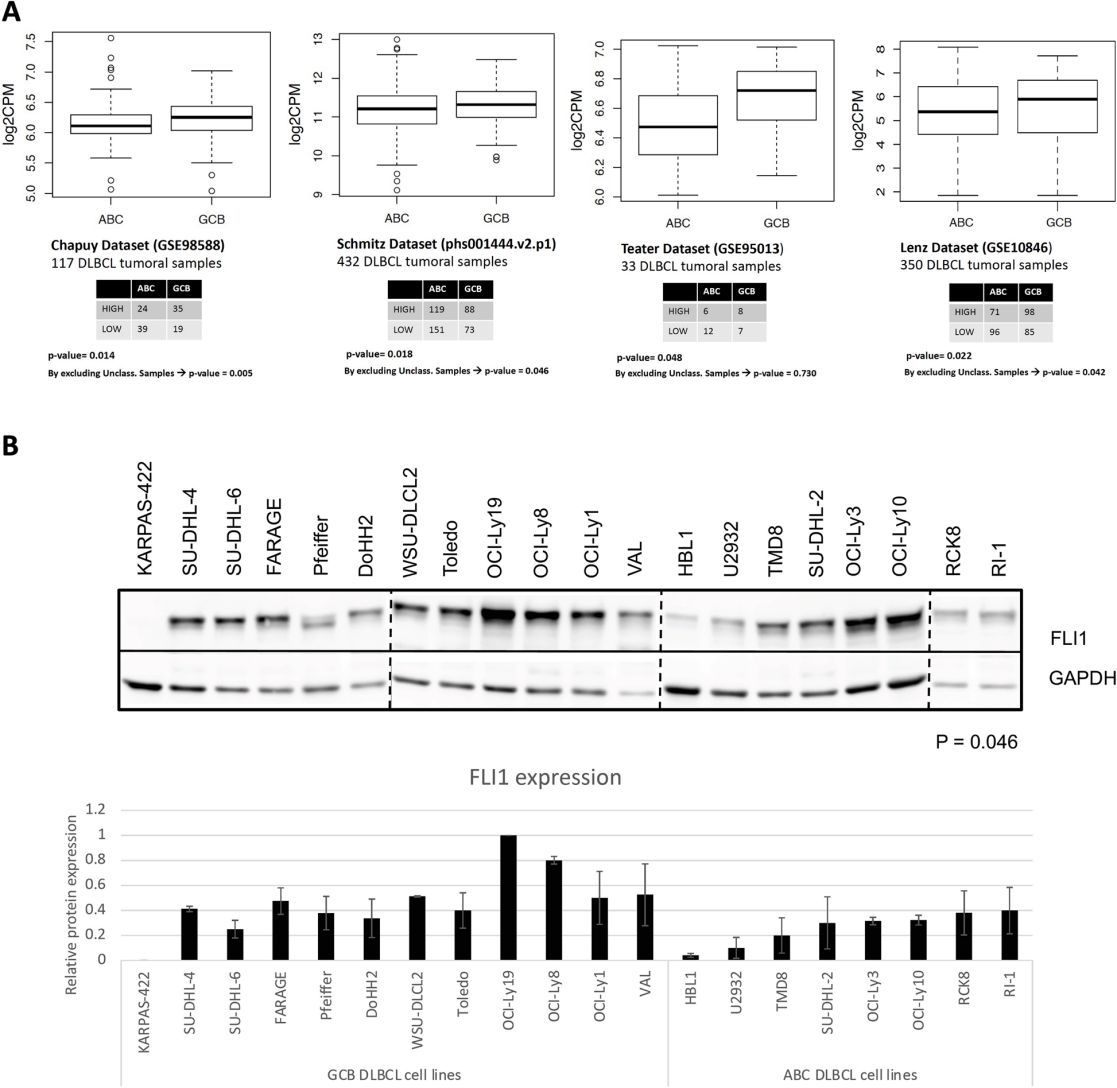
FLI1 expression in DLBCL clinical specimens and cell lines. **A** Differential expression of FLI1 RNA in four datasets comparing GCB DLBCL to ABC DLBCL. In the contingency tables, FLI1 mRNA expression was dichotomized into high and low values using the median as a cut-off. **B** Immunoblot showing protein expression of FLI1, in twelve GCB DLBCL and eight ABC DLBCL cell lines; mouse monoclonal α-GAPDH was used as loading control; quantification of FLI1 protein levels in two replicates (two-tailed T test *P* value = 0.046)

We analyzed the pattern of *FLI1* RNA expression in four publicly available datasets of DLBCL clinical specimens: GSE98588, phs001444.v2.p1, GSE95013 and GSE10846 [5, 6, 21, 22]. High *FLI1* expressors were enriched among GCB (total n. = 414) compared to ABC (total n. = 518) DLBCL samples (*P* < 0.05) (Fig. 1A). A higher FLI1 expression was also observed in the C3 and EZB genetic subclasses enriched in GCB DLBCL compared to the C5 and MCD subclasses enriched in ABC DLBCL (Fig. S1A). In agreement with these findings, FLI1 protein levels were higher in GCB (n. = 12) than ABC DLBCL (n. = 8) derived models (Fig. 1B) (*P* = 0.046). A similar difference, although not statistically significant, was observed in terms of RNA expression (Fig. S1B). Based on these data we focused further experiments on GCB DLBCL, since it appeared to be an optimal model for studying the role of FLI1 in DLBCL.

### Down-regulation experiments by siRNA identify FLI1-regulated genes in GCB DLBCL

**Fig. 2:**
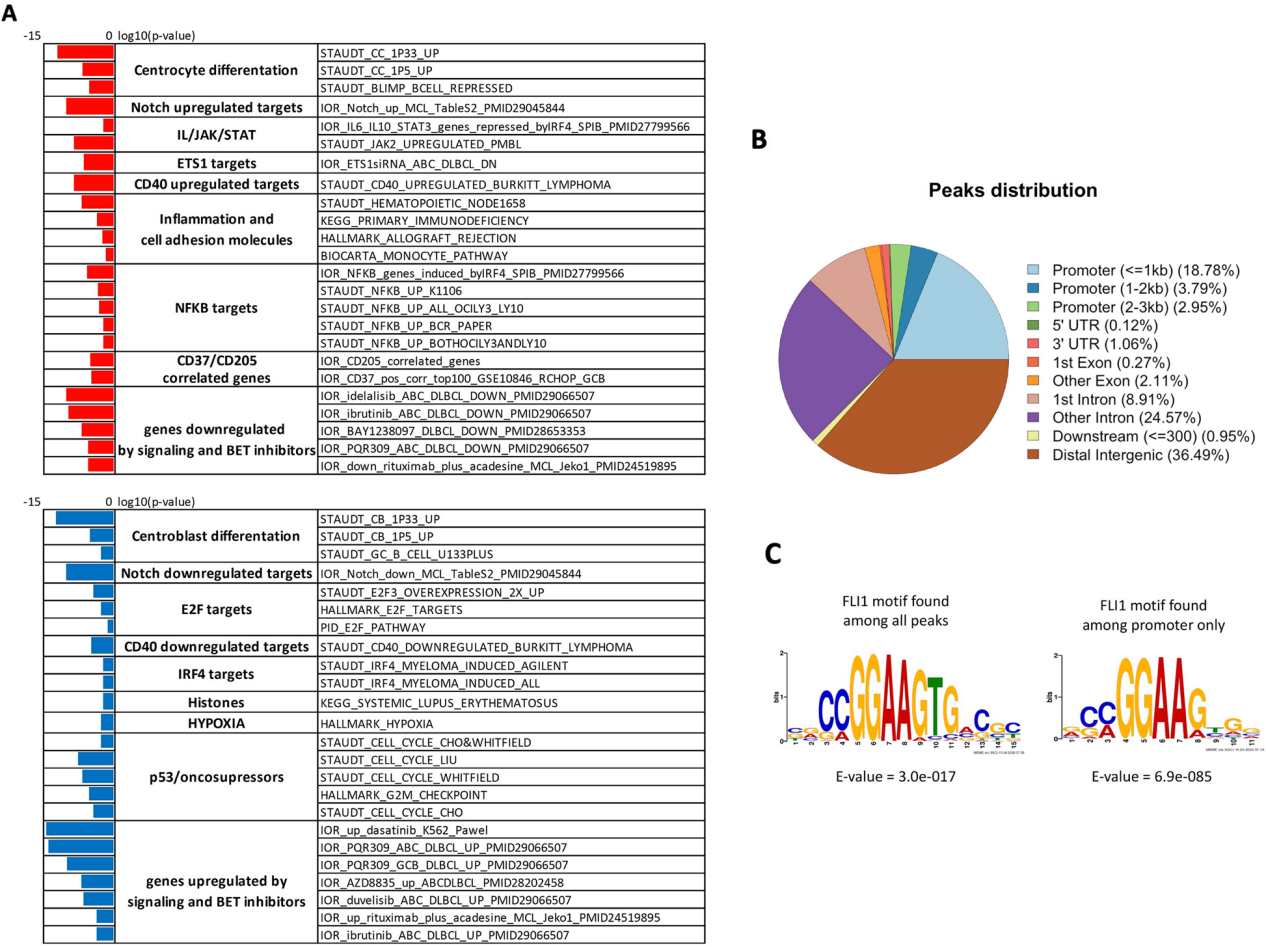
Pathway analysis following FLI1 downregulation and genomic distribution of FLI1 binding sites identified by ChIP-Seq. **A** Summary of pathways enriched in FLI1 up- (red) or downregulated (blue) genes after RNA-Seq, comparing FLI1 knockdown versus control samples in the GCB DLBCL cell lines OCI-Ly1 and VAL (absolute logfold change > 0.1 and adj.*P* < 0.05). Significant g:GOSt annotated pathways/signatures (adjusted *p*-value < 0.05) are grouped into biological processes and sorted by adjusted *p*-value. **B** Distribution of FLI1 binding sites as assessed by ChIP-Seq in both GCB DLBCL cell lines. **C** Consensus binding motif enrichment found with MEME for all 16,865 OCI-Ly1 peaks plus the top 16,865 VAL peaks or for the promoter only OCI-Ly1 plus VAL peaks

To identify genes and pathways regulated by FLI1 in GCB DLBCL, we performed RNA-Seq in two cell lines, with three replicates each, derived from GCB DLBCL (OCI-Ly1 and VAL) after FLI1 downregulation by siRNAs (Fig. S2A-B). FLI1 knockdown affected the transcriptome of the cells, and a supervised analysis followed by functional characterization of the affected transcripts showed that FLI1 positively regulated genes involved in NF-κB and BCR signaling, the CD40 pathway, ETS1 and NOTCH targets, and genes repressed by BLIMP1 (Fig. 2A; Table S2). E2F-repressed targets and hypoxia-related genes were FLI1 negatively regulated (Fig. 2A; Table S2). FLI1 also positively regulated genes that are downregulated in lymphoma cell lines exposed to signaling inhibitors such as the PI3K delta inhibitor idelalisib, the BTK inhibitor ibrutinib and the BET Bromodomain inhibitors, while negatively controlling genes upregulated by the same compounds (Fig. 2A; Table S2B).

### Identification of FLI1 binding sites in DLBCL cells

To identify FLI1 binding sites across the genome, we performed ChIP-Seq with an anti-FLI1 antibody on the same cell lines used for RNA-Seq. FLI1 binding sites were observed in intergenic regions (37% of the peaks), in intragenic regions (37%) including introns, exons and UTRs, as well as in promoter regions of annotated transcripts (26%) (Fig. 2B and Fig. S3A). The peaks distribution was in agreement with publicly available FLI1 ChIP-Seq data derived from several cancer cell types (Table S3A). Indeed, ChIP-Seq peaks from the two cell lines, including peaks distant from transcriptional starting site (TSS), were enriched for the consensus FLI1 binding motif (Fig. 2C and Fig. S3B). A total of 13,339 peaks were detected in promoter regions within 3 Kb of the transcription start site (TSS), 9223 coming from VAL and 4116 from OCI-Ly1 (Table S3B). Removing duplicates, 7860 transcripts including protein coding genes, non-coding RNA (ncRNA), snoRNA, snRNA and pseudogenes were identified as bearing one or more FLI1 peaks at ChIP-Seq in their promoter regions (Table S3C). Among these, 2791 were common between VAL and OCI-Ly1. Fig. S3C and D show examples of FLI1 ChIP-Seq peaks and their validation by Real-Time qPCR in the promoters of genes including *WEE1*, a known FLI1 target.

### Integration of ChIP-Seq with RNA-Seq data identifies direct promoter targets of FLI1

**Fig. 3:**
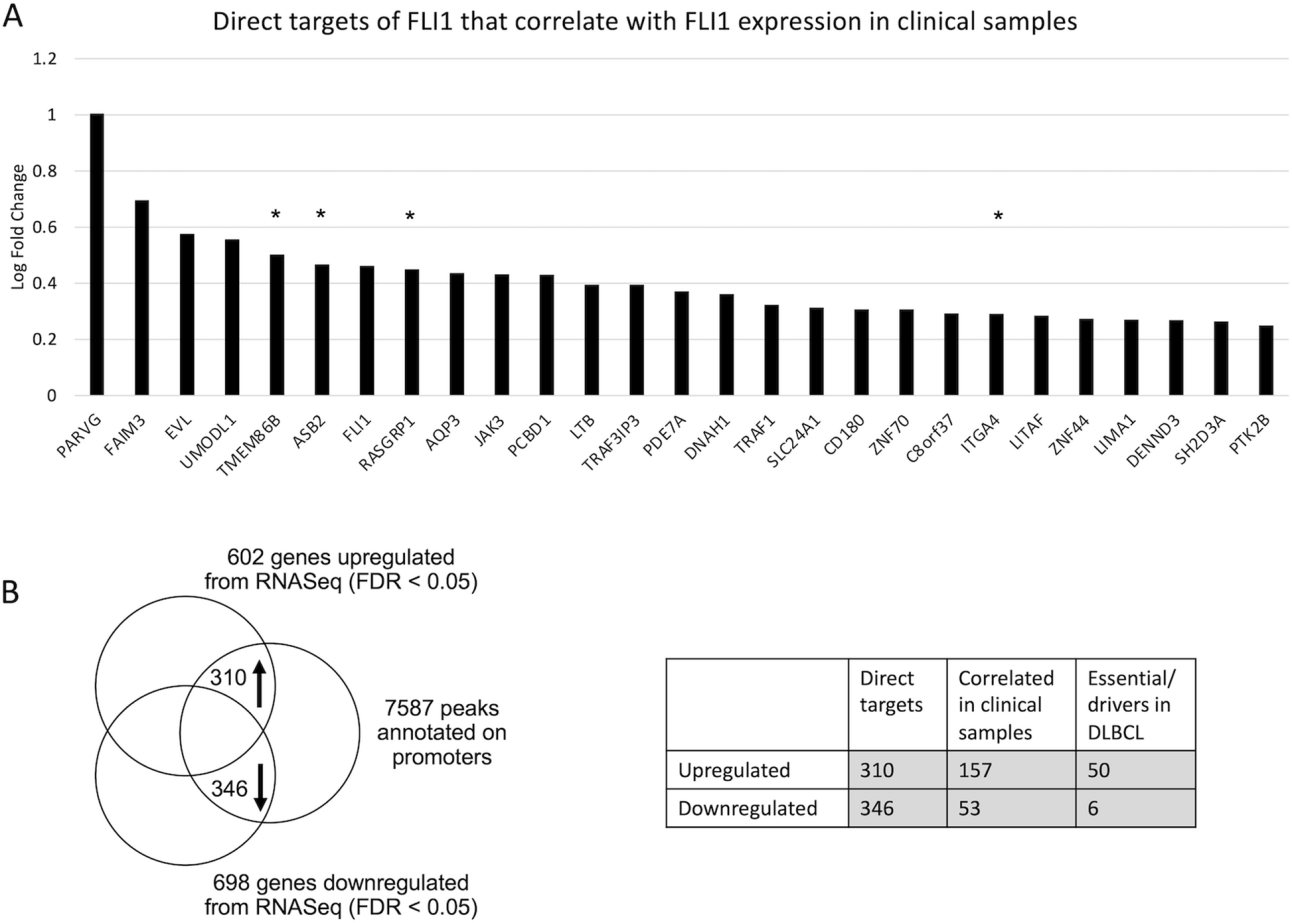
Integration of RNA-Seq with ChIP-Seq and clinically correlated genes. **A** Integration of RNA-Seq upregulated genes and downregulated genes with ChIP-Seq data. The table shows the number of genes obtained after integration with clinically correlated genes (Fig. S3A for intersection) and drivers (Fig. S3B for intersection). **B** FLI1 direct targets with log fc > 0.25, direct targets of FLI1 whose expression correlates with FLI1 in GCB DLBCL clinical specimens; *, DLBCL drivers according to Reddy et al. [36]

To discriminate between primary and secondary FLI1 target genes, we overlapped the promoter regions determined by ChIP-Seq with RNA-Seq data from FLI1-silenced cell lines. We identified 346 negatively regulated direct targets and 310 positively regulated genes (Fig. 3A; Table S4A). This suggested that many of the gene sets modulated by FLI1 downregulation resulted from direct regulation of their component genes by the transcription factor. Among the positively regulated direct targets there were transcripts involved in inflammation (CD40 pathway), MYC targets, NF-κB and BCR signaling, ETS1 targets, NOTCH signaling, genes repressed by BLIMP1 (involved in centrocyte differentiation) and ribosomes. FLI1 negatively regulated direct targets were enriched for genes involved in the negative regulation of mitotic cell cycle and hypoxia (Table S4B). To extend our findings to clinical specimens, we integrated our results with a publicly available gene expression dataset of GCB DLBCL cases (GSE10846) [22]. We confirmed 53 FLI1 downregulated genes as negatively correlated to FLI1 and 157 FLI1 upregulated genes as positively correlated (Fig. S4A; Table S5A) in clinical samples. A CRISPR-Cas9 screen performed in GCB DLBCL cell lines had defined a series of DLBCL essential genes, based on the fact that their silencing resulted in significantly decreased cell fitness in at least one DLBCL cell line [38]. Here, we observed that 50 out of the 157 FLI1 upregulated transcripts belonged to these essential genes, underlining the importance of this transcription factor (Fig. S4B; Table S5B). Figure 3B shows direct targets of FLI1 that were also correlated with FLI1 expression in clinical specimens and had an absolute fold change > 2.5 after downregulation.

### Integration of ChIP-Seq and capture hi-C (C-HiC) with RNA-Seq data identifies distal direct FLI1 targets

As seen for other transcription factors [39], there were peaks located further than 3 kb from the closest TSS. By using a Capture HiC (C-HiC) map of enhancer-promoter pairs obtained in an EBV-transformed lymphoblastoid cell line [36], FLI1-bound enhancers were associated to 18,898 candidate target genes; 4,600 of these were common to promoter peaks (Fig. S5A; Table S6). Of these, 447 were also negatively regulated and 390 positively regulated by FLI1 based on the downregulation experiments. A role for FLI1 in the regulation of these genes was also sustained by correlation analyses with FLI1 expression in clinical specimens in which 193 positively regulated and 328 negatively regulated genes were identified. These comprised GCB DLBCL essential genes (n. = 53 and n. = 13, respectively) (Fig. S5B; Table S7). Excluding genes that also had a binding peak in their promoter regions, we identified 17 positively and 13 negatively regulated distal direct FLI1 targets (Table S7C).

### ASB2 is a target of FLI1 and regulates the NF-κB pathway in GCB DLBCL

**Fig. 4:**
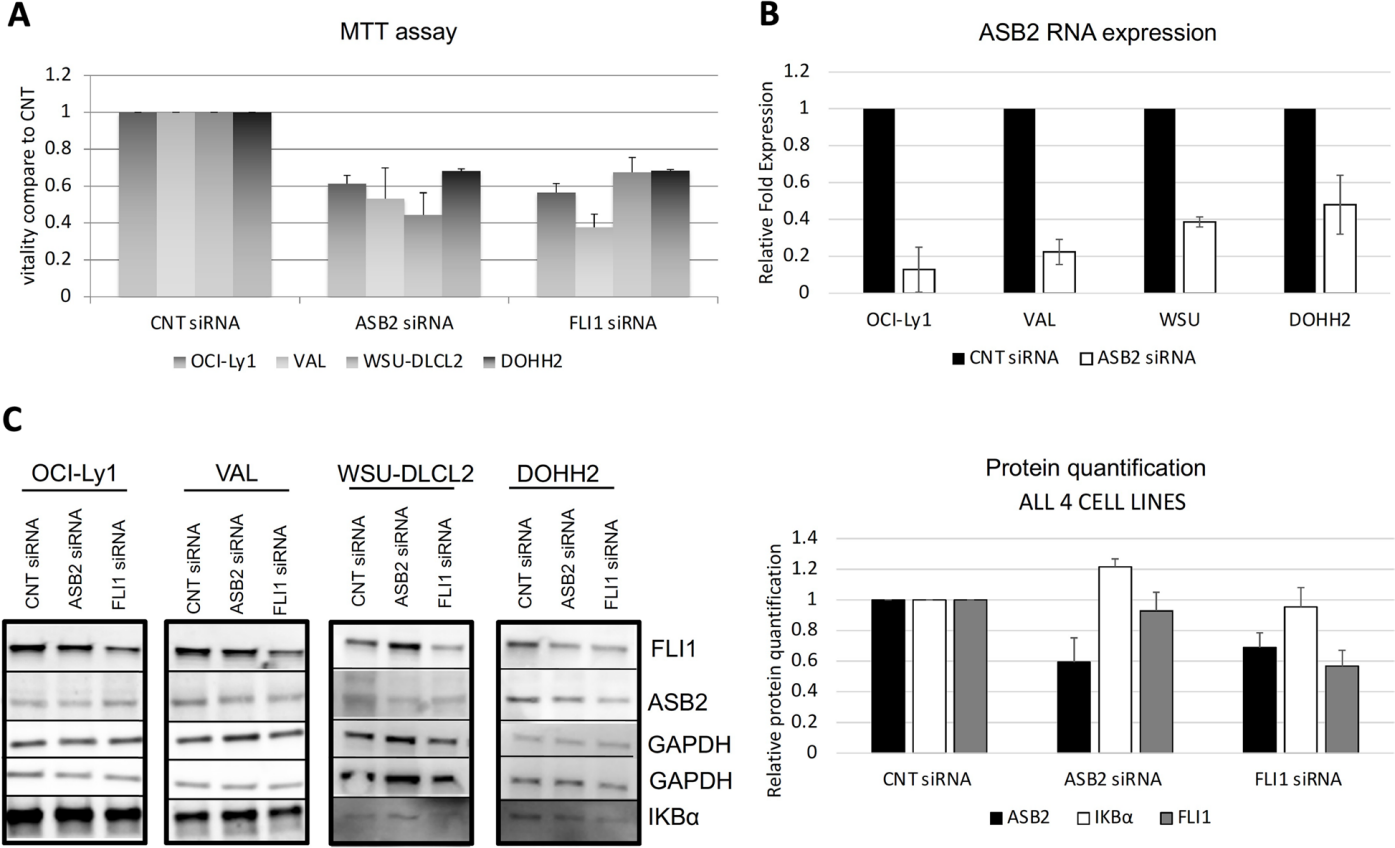
ASB2 downregulation in DLBCL cell lines harvested 72 h after nucleofection. **A** MTT assay for DLBCL cell lines nucleofected with either 500 nM control (CNT) siRNA, FLI1 siRNA or ASB2 siRNA. **B** Normalized (to GAPDH) relative mRNA expression of ASB2 from CNT siRNA and ASB2 siRNA treated cells. **C** Immunoblot and its quantification showing protein expression of ASB2 and IκBα in DLBCL CNT siRNA, FLI1 siRNA and ASB2 siRNA treated cells. Mouse monoclonal α-GAPDH was used as loading control. For each figure two replicates were performed for each cell line

**Fig. 5:**
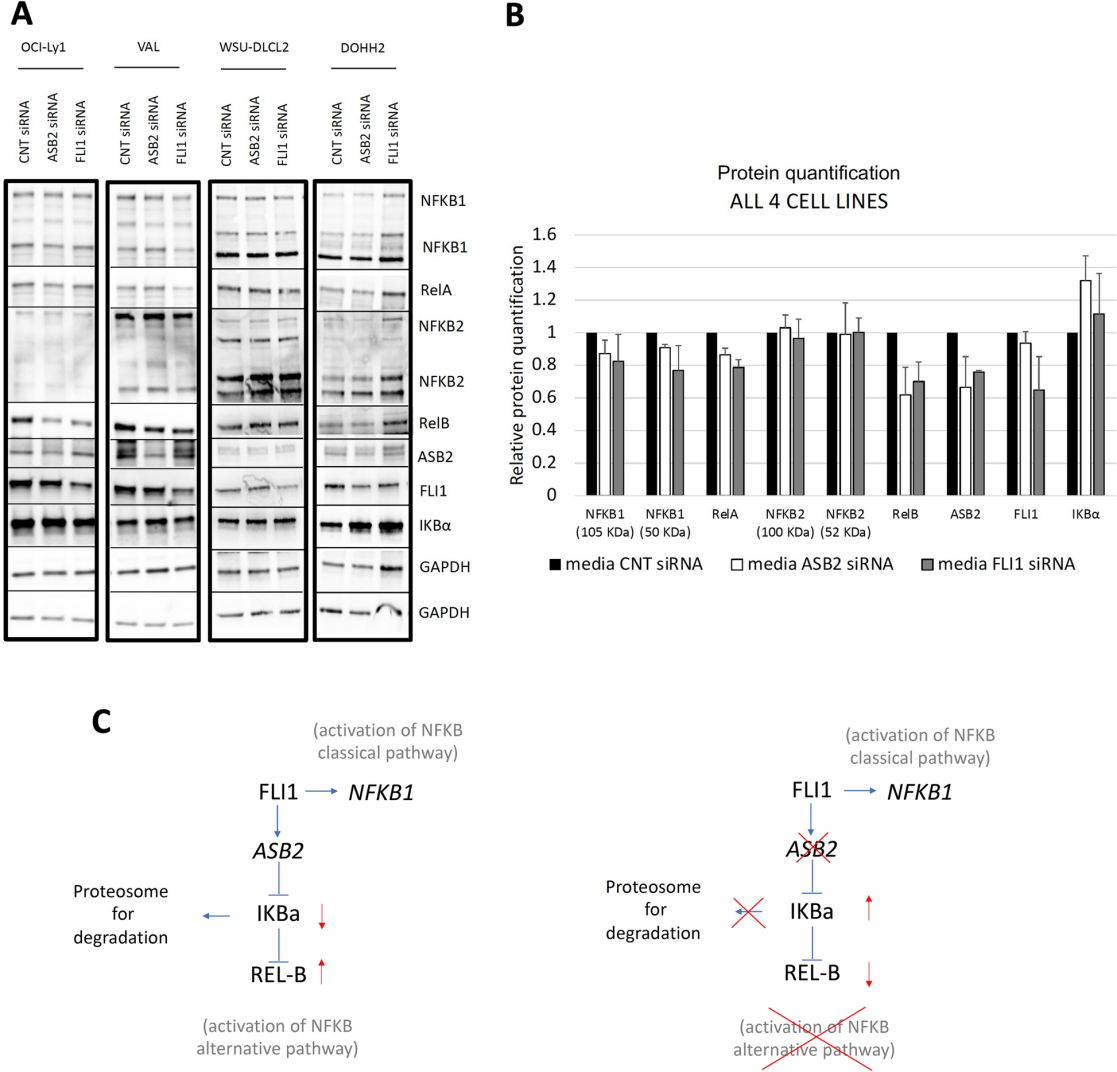
ASB2 downregulation in DLBCL cell lines harvested 48 h after nucleofection. **A** Immunoblots for NF-κB related genes after ASB2 and FLI1 downregulation and **B** quantification of protein bands. **C** Schema of the proposed mechanism of FLI1 transcriptional regulation of NF-κB1 and ASB2 genes, that respectively contribute to the activation of the classical and alternative NF-κB pathway. For each figure two replicates were performed for each cell line

ASB2 expression levels were reduced after FLI1 downregulation (Fig. S6A) and had FLI1 binding sites in both its promoter region (Fig. S2D) and distal enhancer regions (Fig. S6B). ASB2, regulated by Notch1, promotes NF-κB activation in T-cell acute lymphoblastic leukemia [40], and based on a genetic screen, it is a putative DLBCL essential gene [38]. Accordingly, *ASB2* gene downregulation was toxic in four GCB DLBCL cell lines, bearing *BCL2* translocation (OCI-Ly1, WSU-DLCL2) or concomitant *BCL2/MYC* translocation (VAL, DOHH2) (Fig. 4A and B). We then evaluated the status of NF-κB pathway activation by assessing the expression level of the NF-κB inhibitor, IκBα. IκBα was upregulated 72 h after ASB2 downregulation compared to siRNA CNT and siRNA FLI1 (Fig. 4C; Fig. S7A). At 48 h, IκBα upregulation was again stronger after ASB2 than FLI1 downregulation (Fig. 5A and B; Fig. S7B), suggesting an important role for ASB2 in regulating IκBα. In addition to increasing IκBα, ASB2 downregulation resulted in a strong reduction of RelB protein, with no change in NF-κB2 levels (Fig. 5A and B; Fig. S7B). This reduction was also observed after FLI1 downregulation with a lesser extent (Fig. 5A and B; Fig. S7B). These results suggest that FLI1 upregulates the transcription factor of the alternative NF-κB pathway, RelB, via ASB2 and downregulates IκBα in GCB DLBCL. Downregulation of FLI1, but not ASB2, caused reduction of NF-κB1 and RelA protein levels (Fig. 5A and B; Fig. S7B), paired with decreased NF-κB1 RNA levels at RNA-Seq and a reduction of NF-κB1 nuclear translocation validated by immunofluorescence (Fig. S7). This indicates that FLI1 regulates the classical NF-κB pathway at the transcriptional level in GCB DLBCL. Indeed, both classical and alternative NF-κB pathway gene signatures were enriched among FLI1-regulated genes. In conclusion, FLI1 regulates the NF-κB pathway in GCB DLBCL (Fig. 5C).

## Discussion

Of the two ETS transcription factors mapped within the 11q24.3 locus that is recurrently gained in approximately 25% of DLBCLs, ETS1 is more expressed in ABC than in GCB DLBCL [8, 9]. Here, using large expression datasets we determined that FLI1 is expressed at a higher level in GCB than ABC DLBCL and defined the network of genes regulated by this transcription factor in the GCB DLBCL subtype.

Transcriptome analysis after FLI1 downregulation showed that FLI1 regulates important biological pathways. Integration of the identified binding sites with RNA-Seq from cell lines after FLI1 downregulation allowed the identification of putative direct targets of FLI1. Transcripts negatively regulated by FLI1 included tumor suppressor genes involved in the negative regulation of mitotic cell cycle and hypoxia. Among the FLI1 positively regulated targets we found genes annotated as MYC targets and members of BCR, CD40, TNFα and IL2 signaling pathways. Of note, direct targets of FLI1 overlapped with genes regulated by ETS1, the other transcription factor gained within the 11q24.3 locus in DLBCL [9], suggesting a functional convergence of the ETS factors. In particular, FLI1 positively regulated transcripts such as *CXCR5, CD40, CD79A, NF-κB1, ITGA4, FAIM3, DDX21, AATF, RASGRP1, LYN, JAK3* and *TRAF1/4/5*. Conversely, *CDKN1B, EZR, E2F7* and *TFDP2* were among the negatively regulated genes. Our results in cell lines were sustained by findings in clinical specimens: FLI1 positively regulated targets in cell lines overlapped with genes that positively correlated with the expression levels of the transcription factor in GCB DLBCL samples. Moreover, we took advantage of a genome-wide CRISPR-Cas9 screen of DLBCL cell lines [38] and identified a series of DLBCL driver genes that appeared as direct FLI1 targets, including ASB2 (ankyrin repeat-containing protein with a suppressor of cytokine signaling box 2). The ASB2 protein is a subunit of a multimeric E3 ubiquitin ligase complex, and the classic function of ASB2 is to target specific proteins for ubiquitination and degradation by the proteasome [41, 42]. Our results suggested that FLI1 regulates the expression of ASB2, which in turn downregulates IκBα, an important inhibitor of the NF-κB pathway. Activation of the NF-κB pathway is a recurrent phenomenon in DLBCL, and usually correlates with a more aggressive clinical course [1–3, 5, 6]. Although NF-κB signaling is more commonly associated with the ABC phenotype, it is also active in half of GCB DLBCL cases [4]. Moreover, recurrent somatic mutations in the *NFKBIA* gene, encoding IκBα, are associated with a poor outcome in GCB DLBCL cases [38]. Finally, based on the Wright et al. classification [43], NF-κB is deregulated by IκBα (*NFKBIA*) inactivation also in some GCB DLBCL belonging to ST2 tumors [44]. The NF-κB protein RelB, forms a dimer with the processed p52 form of NF-κB2 and acts as a transcription factor of the alternative pathway. Ablation of both RelB and NF-κB2 results in the collapse of established germinal centers [45]. In dendritic cells, RelB does not promote cell activation by dimerizing with p52, effector of the non-canonical NF-κB pathway, but instead dimerizes with the NF-kB1 protein p50. The resulting RelB-p50 heterodimer is regulated by the canonical IκBs, IκBα and IκBε [46]. In GCB DLBCL cells, FLI1 positively regulated p50 (NF-κB1) and ASB2 at the transcriptional level. ASB2 downregulation was followed by increase of the NF-κB negative regulator IκBα, and downregulation of RelB levels. The mechanism we observed in GCB DLBCL cell lines is supported by the reported interactions of ASB2 with IκBα [40] and of the latter with RelB [47–50], although the exact mechanisms need to be fully elucidated. Our data suggest that FLI1 and ASB2 should be further explored in the context of therapeutic targeting NF-κB in GCB DLBCL, including double-hit lymphomas.

## Conclusion

In conclusion, the transcription factor FLI1 was expressed at higher levels in GCB than ABC DLBCL and it directly regulated a network of biologically crucial genes and processes. We identified a mechanism of NF-κB activation mediated by a novel direct target of FLI1, ASB2, which downregulated IκBα and upregulated RelB in GCB DLBCL. ASB2 and FLI1 inhibition represents a potential novel therapeutic approach for GCB DLBCL.

## Supplementary Information

**Additional file 1.**

## Data Availability

All data are available at the National Center for Biotechnology Information (NCBI) Gene Expression Omnibus (GEO) (http://www.ncbi.nlm.nih.gov/geo) database (GSE157191).

## Abbreviations

AATF: Apoptosis Antagonizing Transcription Factor
ABC DLBCL: Activated B-cell-like DLBCL
ASB2: Ankyrin Repeat And SOCS (suppressor of cytokine signaling) Box-Containing Protein 2
BCL2: B-cell lymphoma
BCR: B-Cell Receptor
BLIMP1: B-lymphocyte-induced maturation protein 1
BTK: Bruton’s tyrosine kinase
C-HiC: Capture high throughput chromosome conformation
cDNA: Complementary DNA
ChIP: Chromatin Immunoprecipitation
DEPC: Diethyl pyrocarbonate
DLBCL: Diffuse Large B-cell Lymphoma
E2F: E2 factor
EBV: Epstein-Barr virus
ECL: Enhanced chemiluminescence
EDTA: Ethylenediaminetetraacetic acid
ETS1: ETS Proto-Oncogene 1
EWSR1: Ewing sarcoma breakpoint region1
FC: Fold Change
FDR: False Discovery Rate
FLI1: Friend Leukemia Insertion 1
GAPDH: Glyceraldehyde 3-phosphate dehydrogenase
GCB DLBCL: Germinal Center B-cell-like DLBCL
GEO: Gene Expression Omnibus
GRCh37: Genome Reference Consortium Human Build 37
HCl: Hydrochloric acid
Kb: Kilobase
MRL/lpr: Murphy Roths large/ lymphoproliferative
MTT: 3-(4,5-dimethylthiazol-2-yl)-2,5-diphenyltetrazolium bromide
NaCl: Sodium chloride
NaHCO3: Sodium Bicarbonate
NCBI: National Center for Biotechnology Information
ncRNA: Non-coding RNA
NF-κB: Nuclear Factor Kappa B
NFKBIA: Nuclear factor-kappa-B-inhibitor alpha
PBS: Phosphate-buffered saline
PI3K: Phosphoinositide 3-kinases
qRT-PCR: Quantitative Real-Time polymerase chain reaction
RASGRP1: RAS guanyl-releasing protein 1
SDS: Sodium dodecyl sulfate
siRNA: Small interfering RNA
STR: Short Tandem Repeat
TBST: Tris-buffered saline with 0.1% Tween
TSS: Transcription start site
