# Supplementary material for: ASB2 is a direct target of FLI1 that sustains NF-κB pathway activation in germinal center-derived diffuse large B-cell lymphoma
Source: J Exp Clin Cancer Res. 2021 Nov 11;40:357. doi: 10.1186/s13046-021-02159-3 (PMC8582153; doi:10.1186/s13046-021-02159-3)
Supplement: Supplementary file 1 — Additional file 1. [file 13046_2021_2159_MOESM1_ESM.zip › Sartori et al - revised suppl material.pdf]

ASB2 is a direct target of FLI1 that sustains NF-κB pathway activation in germinal center-derived diffuse large B-cell lymphoma

Giulio Sartori [1], Sara Napoli [1], Luciano Cascione [1,2], Elaine Yee Lin Chung [1], Valdemar Priebe [1], Alberto Jesus Arribas [1,2], Afua Mensah [1], Michela Dall'Angelo [1,2,3], Chiara Falzarano[1], Laura Barnabei[1], Mattia Forcato [4], Andrea Rinaldi [1], Silvio Bicciato [4], Margot Thome [5] and Francesco Bertoni [1,6].

[1] Institute of Oncology Research, Faculty of Biomedical Sciences, USI, Bellinzona, Switzerland; [2] Swiss Institute of Bioinformatics, Lausanne, Switzerland; [3] Department of Computer Science, University of Verona, Verona, Italy; [4] Dept. of Life Sciences, University of Modena and Reggio Emilia, Modena, Italy; [5] Department of Biochemistry, University of Lausanne, Epalinges, Switzerland; [6] Oncology Institute of Southern Switzerland (IOSI), Bellinzona, Switzerland.

Supplementary materials

Supplementary Figure S1. FLI1 expression in DLBCL clinical specimens and cell lines. **(A)** Differential expression of FLI1 RNA in GSE98588 and phs001444.v2.p1 based on genetic subtypes. **(B)** FLI1 RNA levels expression in GCB versus ABC cell lines considering 19 cell lines presented in FIG.1B.

FIG.S1A

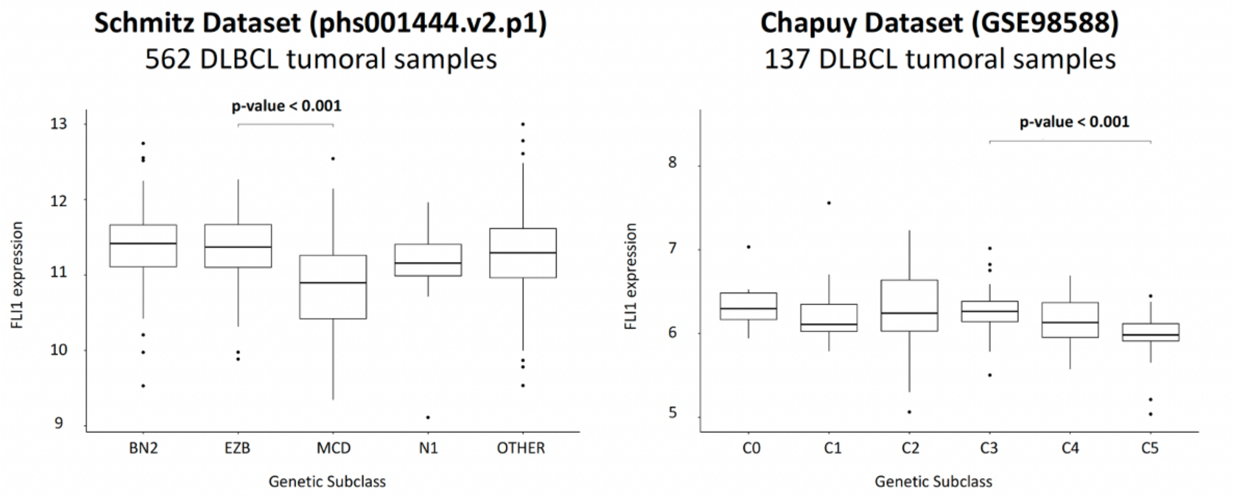

FIG.S1B

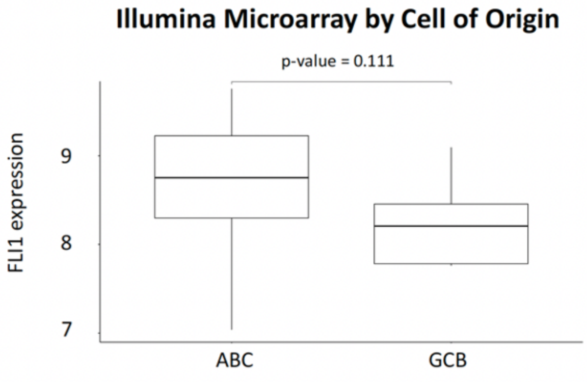

Supplementary Figure S2. FLI1 downregulation in GCB DLBCL cell lines harvested 48 hrs after nucleofection with either 500nM CNT siRNA or FLI1 siRNA. (A) Normalized (to GAPDH) relative mRNA expression of FLI1 from CNT siRNA and FLI1siRNA treated cells. (B) Immunoblot showing protein expression of FLI1 in CNT siRNA and FLI1 siRNA treated cells and (C) its protein quantification. Mouse monoclonal α-GAPDH was used as loading control. Three different replicates are shown.

FIG.S2A

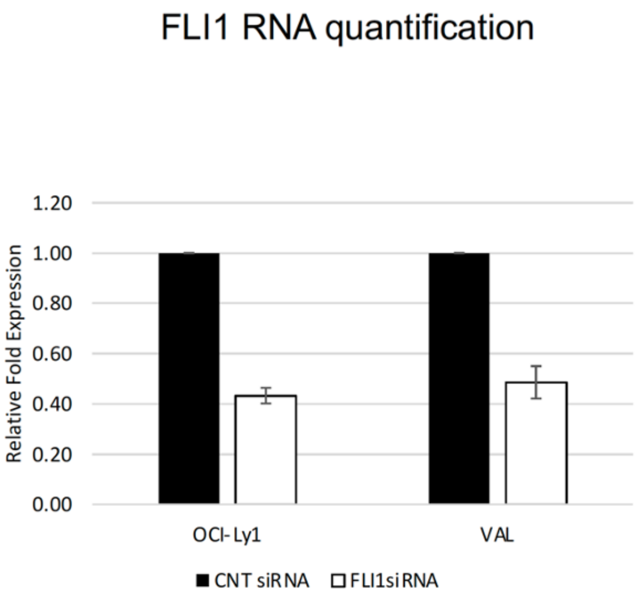

FIG.S2C

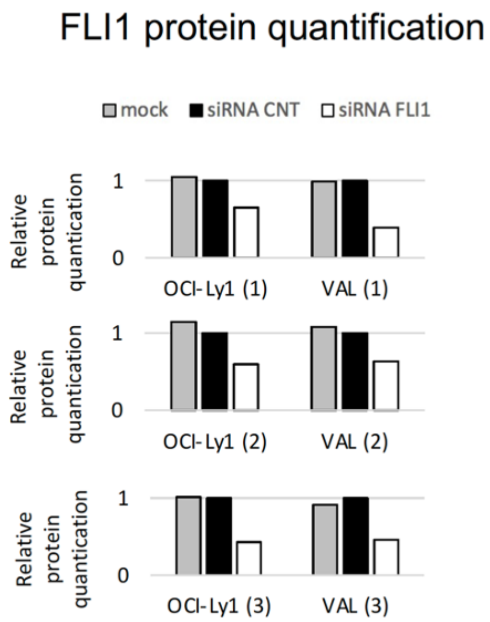

FIG.S2B

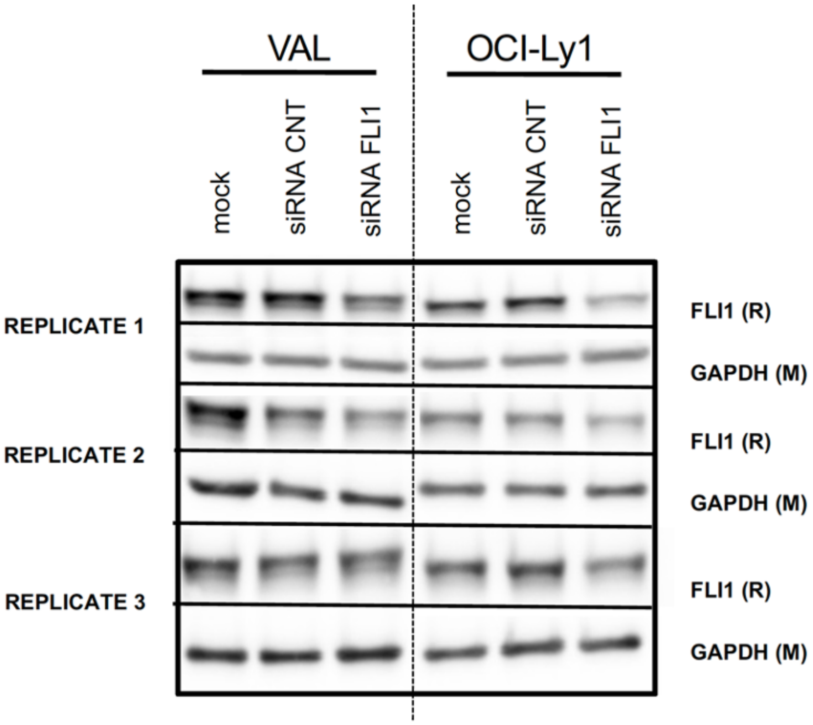

Supplementary Figure S3. FLI1 ChIP-Seq in GCB DLBCL cell lines. (A) Distribution of FLI1 binding sites as assessed by ChIP-Seq in OCI-Ly1 and VAL cell lines. **(B)** MEME-ChIP using vertebrates' database for all 16865 OCI-Ly1 peaks and the top 16865 VAL peaks or for the promoter only OCI-Ly1 and VAL peaks. **(C)** Example of FLI1 binding in the promoter region of some genes as assessed by ChIP-Seq in OCI-Ly1 (upper plot) and VAL (lower plot). FLI1 binding (in green) is overlapped with binding obtained with negative control input DNA (in yellow). **(D)** Validation by real-time PCR of ChIP-Seq data showing FLI1 binding in several promoter regions. The figure shows the relative quantification of promoter regions bound by FLI1 in OCI-Ly1 and VAL. GAPDH amplification was used as a negative control.

FIG.S3A

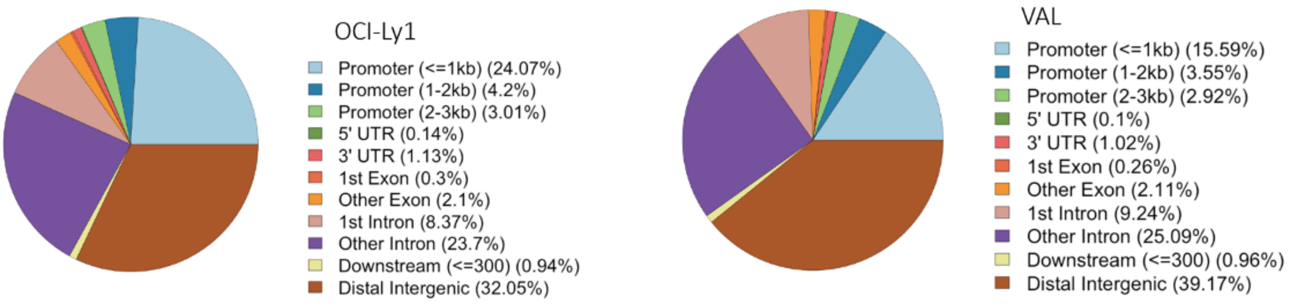

FIG.S3B

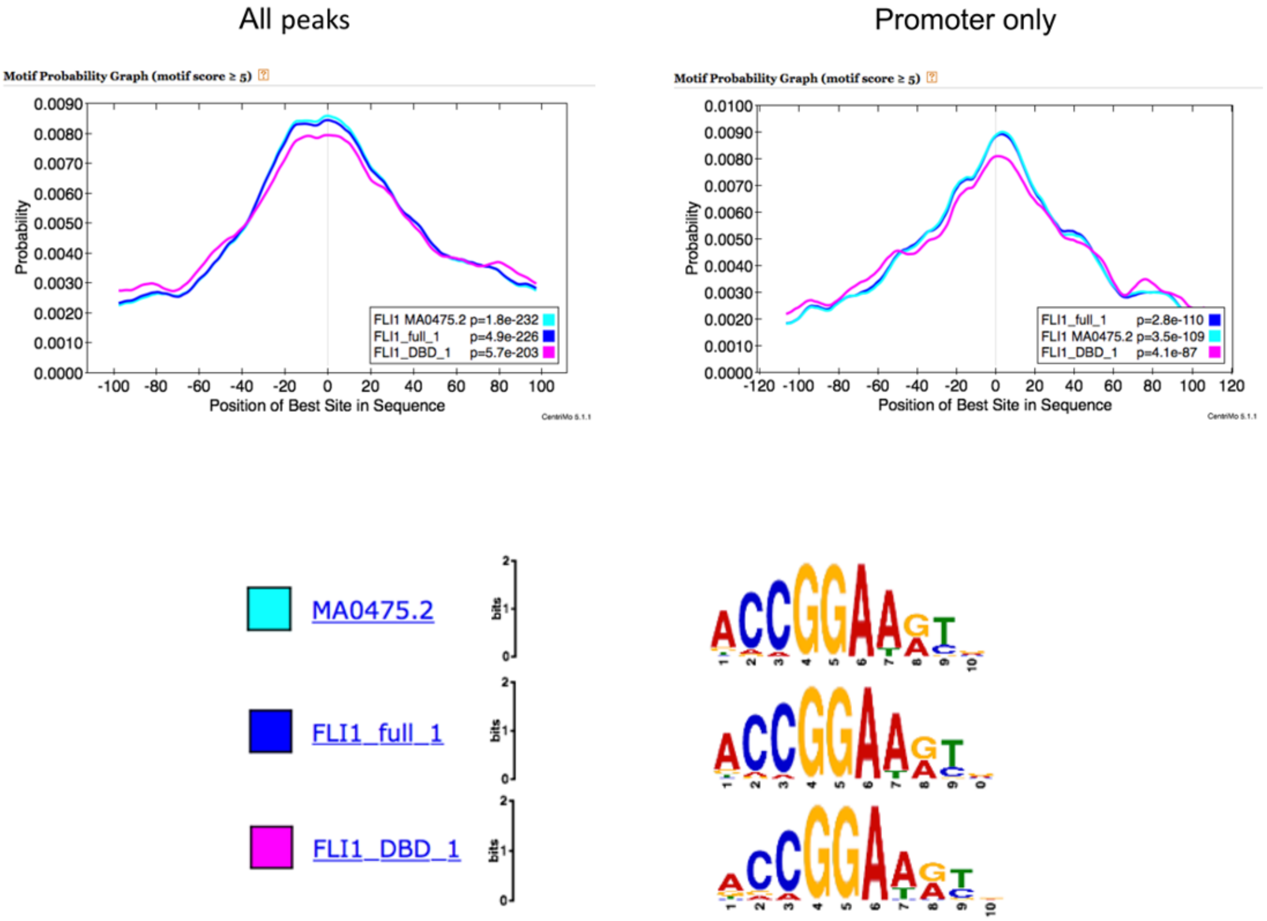

Supplementary Figure S3. FLI1 ChIP-Seq in GCB DLBCL cell lines. (A) Distribution of FLI1 binding sites as assessed by ChIP-Seq in OCI-Ly1 and VAL cell lines. **(B)** MEME-ChIP using vertebrates' database for all 16865 OCI-Ly1 peaks and the top 16865 VAL peaks or for the promoter only OCI-Ly1 and VAL peaks. **(C)** Example of FLI1 binding in the promoter region of some genes as assessed by ChIP-Seq in OCI-Ly1 (upper plot) and VAL (lower plot). FLI1 binding (in green) is overlapped with binding obtained with negative control input DNA (in yellow). **(D)** Validation by real-time PCR of ChIP-Seq data showing FLI1 binding in several promoter regions. The figure shows the relative quantification of promoter regions bound by FLI1 in OCI-Ly1 and VAL. GAPDH amplification was used as a negative control.

FIG.S3C

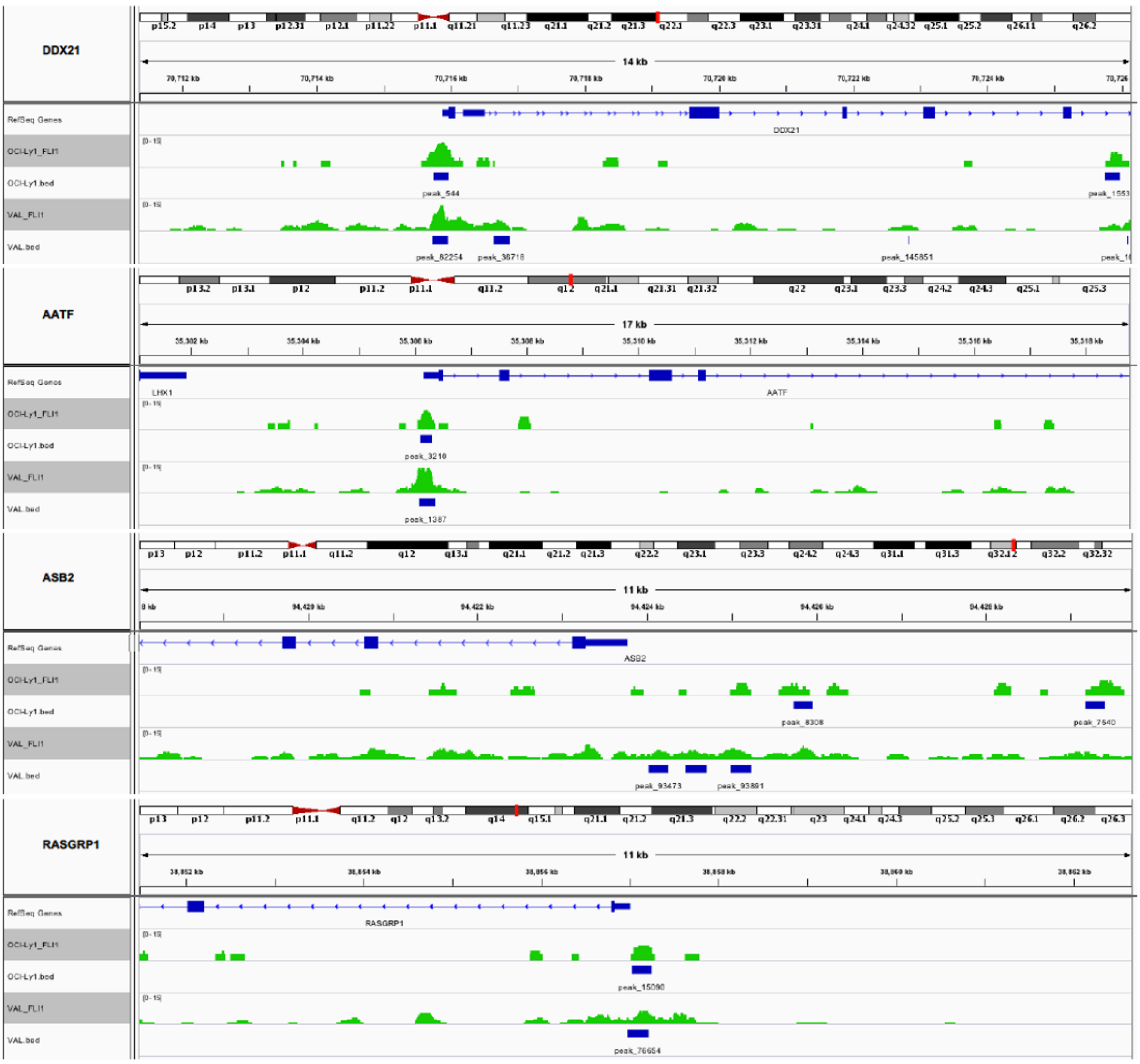

FIG.S3D

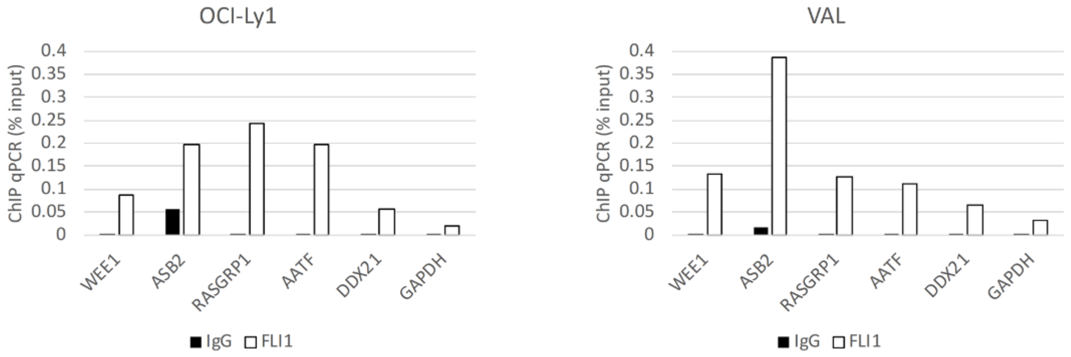

Supplementary Figure S4. Integration of direct targets with FLI1-correlated genes followed by integration with essential genes in DLBCL. (A) Integration of upregulated and downregulated direct targets with FLI1-correlated genes in 183 clinical DLBCL specimens. **(B)** Then we integrated the 157 and 53 overlapped genes from (A) with essential genes in DLBCL according to Reddy et al.[1] The table shows the number of genes obtained after the different integrations.

FIG.S4A

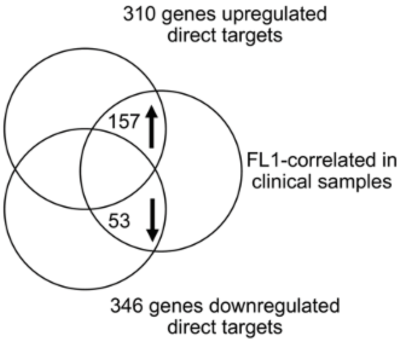

FIG.S4B

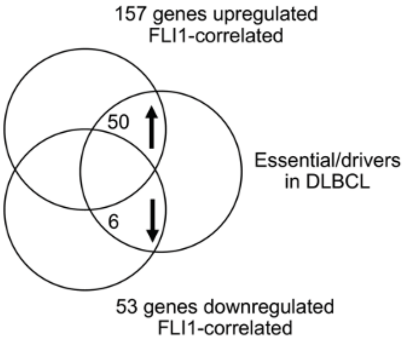

	Direct targets	Correlated in clinical samples	Essential/ drivers in DLBCL
Upregulated	310	157	50
Downregulated	346	53	6

Supplementary Figure S5. C-HiC analysis. (A) Venn diagram showing overlap between FLI1 ChIP-Seq peaks annotated using C-HiC on distal regions, and using HOMER on promoters. **(B)** Integration of transcript identified by supervised analysis from RNA-Seq data (adjusted p-value < 0.05) with FLI1 ChIP-Seq peaks annotated using C-HiC, followed by integration of FLI1-correlated genes in 183 clinical DLBCL specimens and then integration with essential genes in DLBCL. The table shows the number of genes obtained after the different integrations.

FIG.S5A

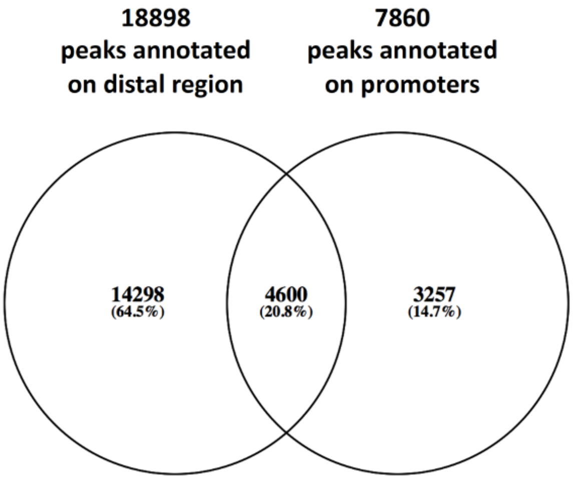

FIG.S5B

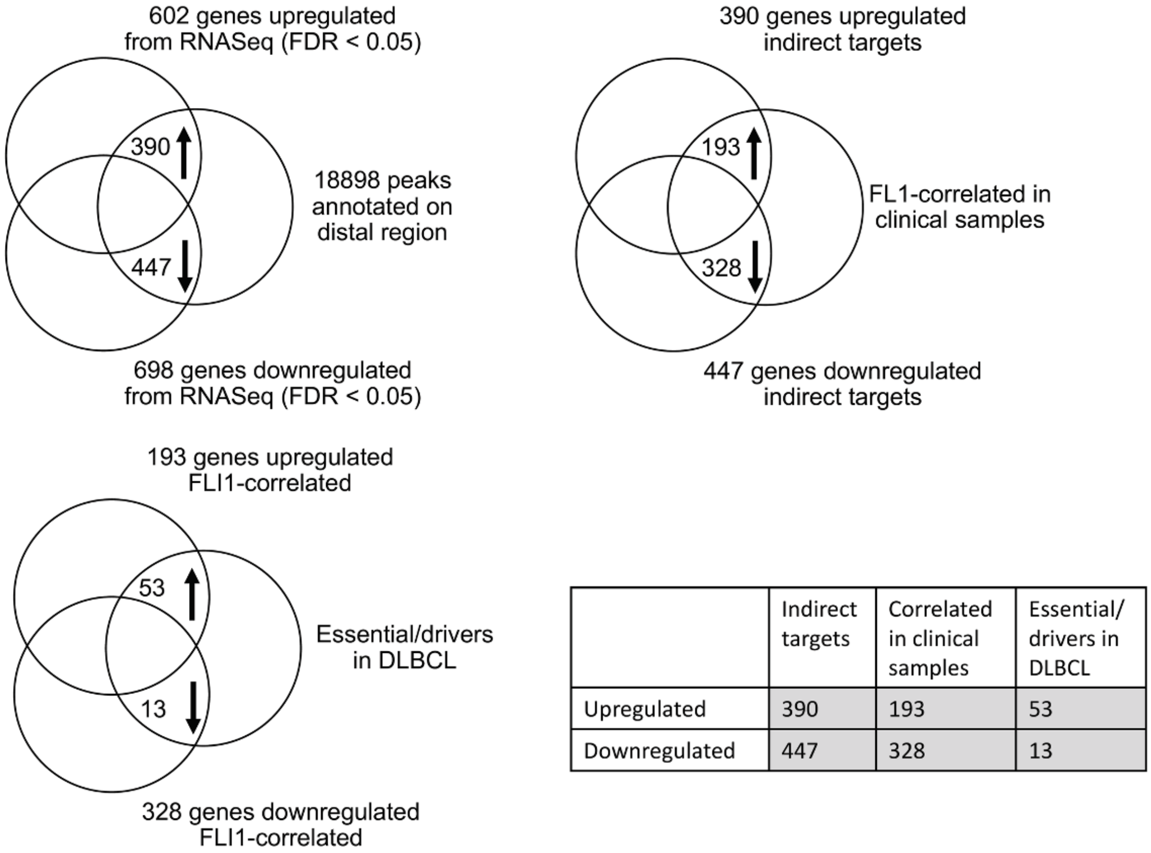

	Indirect targets	Correlated in clinical samples	Essential/ drivers in DLBCL
Upregulated	390	193	53
Downregulated	447	328	13

Supplementary Figure S6. ASB2 is a direct target of FLI1. (A) Normalized (to GAPDH) relative mRNA expression of *ASB2* after FLI1 knockdown with FLI1 siRNA in GCB DLBCL cell lines. Results shown are representative of three individual experiments. n=3; error bars=standard deviation. **(B)** Graph showing FLI1 peaks called by ChIP-Seq (red) located in specific distal regions (green) that interacted with the promoter of *ASB2* (highlighted in yellow). Significant promoter-enhancer interactions are shown as purple arcs.

FIG.S6A

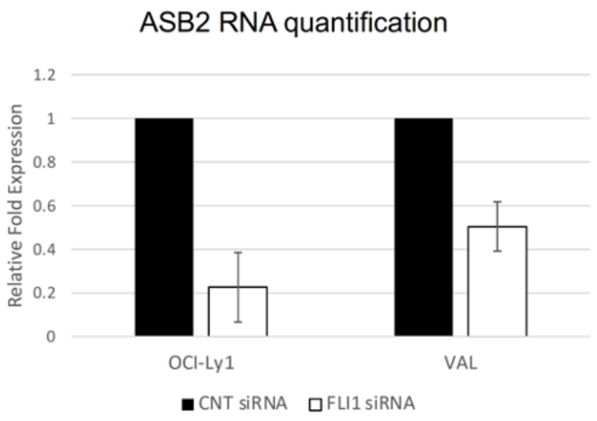

FIG.S6B

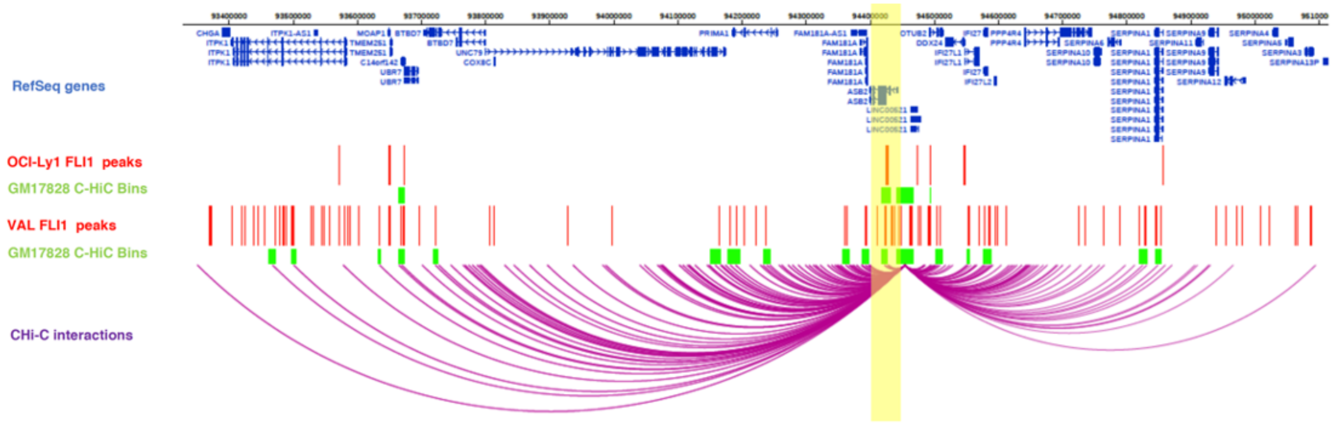

Supplementary Figure S7. ASB2 silencing in DLBCL cell lines harvested 48 and 72 hrs after nucleofection. (A) Second replicate of immunoblots at 48 hrs shown in FIG.4 and its quantification divided per cell lines. **(B)** Second replicate of immunoblots at 72 hrs shown in FIG.5 and its quantification divided per cell lines.

FIG.S7A

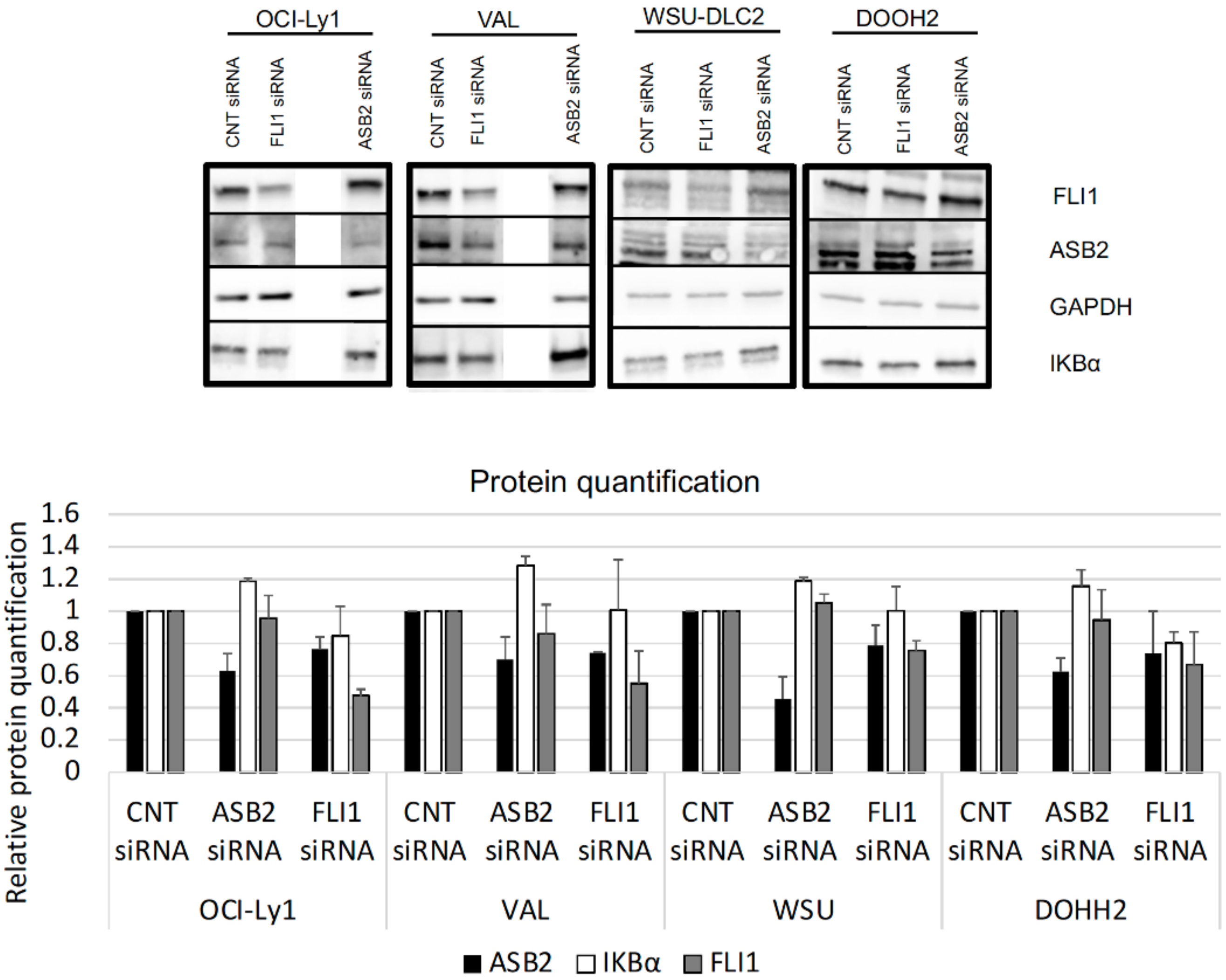

Supplementary Figure S7. ASB2 silencing in DLBCL cell lines harvested 48 and 72 hrs after nucleofection. (A) Second replicate of immunoblots at 48 hrs shown in FIG.4 and its quantification divided per cell lines. **(B)** Second replicate of immunoblots at 72 hrs shown in FIG.5 and its quantification divided per cell lines.

FIG.S7B

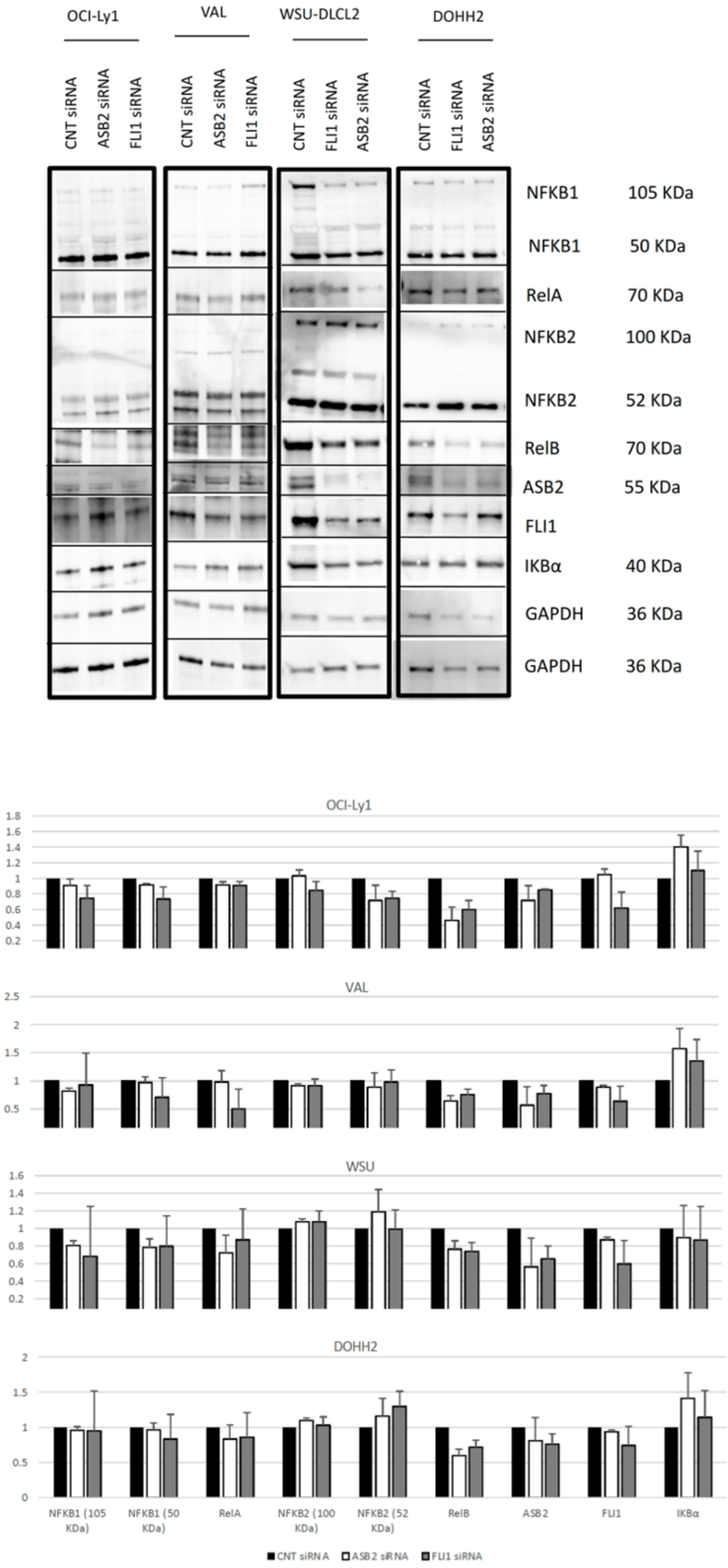

Supplementary Figure S8. Reduced NF-κB1 nuclear translocation and levels after FLI1 down-regulation. (A) Immunofluorescence analysis showing NF-κB1 (red) localization between cytosol and nucleus (blue). BLOCK-iT (green) was used as a control for nucleofection efficiency. 63X oil. **(B)** Zoom in of 7. **(C)** NF-κB1 signal intensity and nuclear quantification.

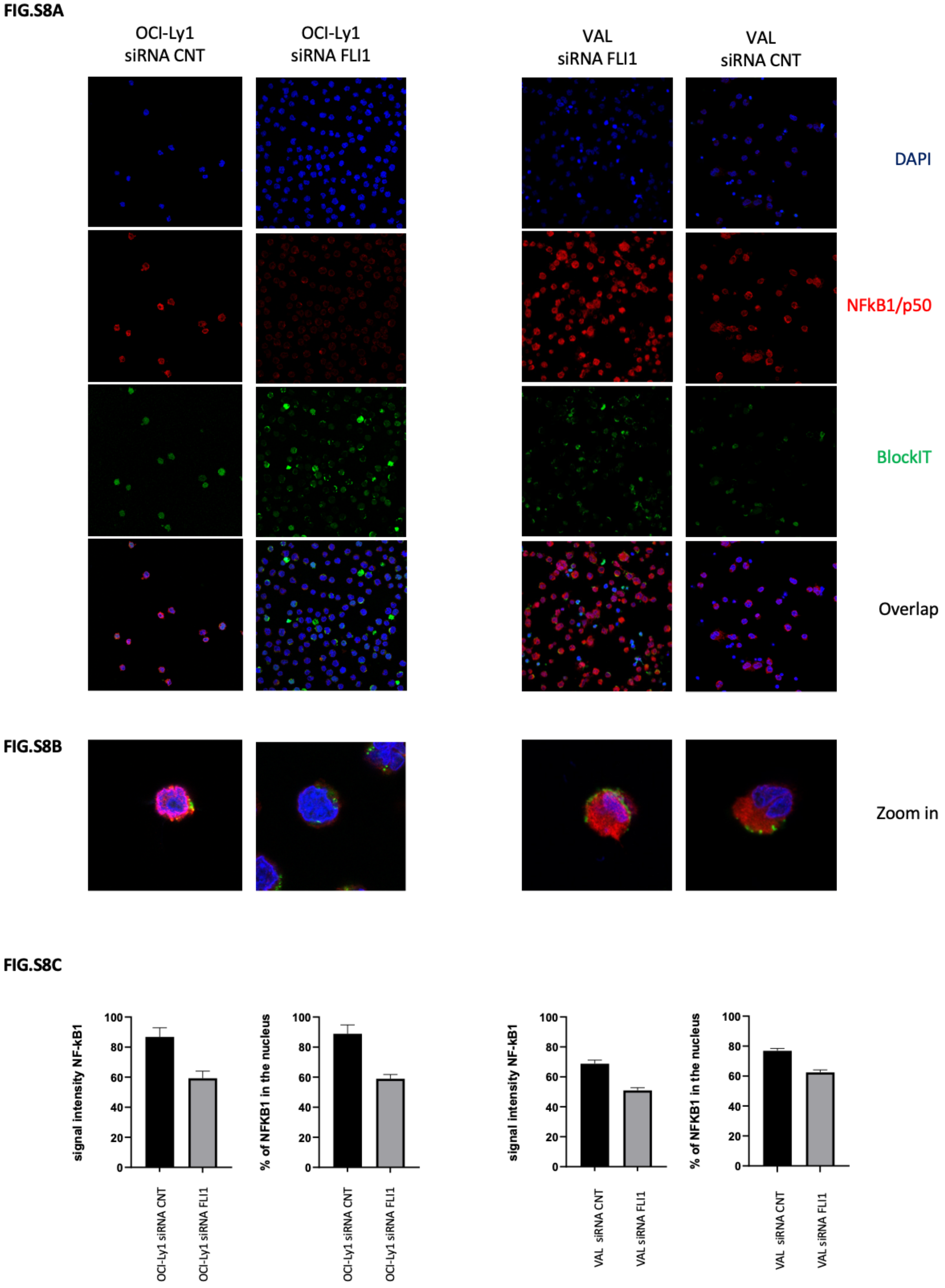

SUPPLEMENTARY TABLES

Supplementary Table 1. List of primers. (A) Primers RT-qPCR and **(B)** Primers ChIP-qPCR

**A)**	**primer Fwd**	**primer Rev**
FLI1	GTGCACAGGGGAGTGAGG	TCACTGGCTGATTGATCCAC
ASB2 isoform 1	CATTGGGCAGGAGGAGTACA	TCTCAGGAGGTGCAGTGGA
ASB2 isoform 2	ACCCGCTTCTCCTATGCAG	CCTTGGAACAAGCCCATTG
GAPDH	GGCTGTGGGCAAGGTCATCCCTGA	TCCACCACCCTGTTGCTGTA
**B)**	primer Fwd	primer Rev
ASB2	GCGACCGCTCAGAGTTACTG	TCTCGCCTGTGATGACTCAG
AATF	CCAATCCCTTCAACCTTCTG	CTCTGGACCGGCCACTTC
RASGRP1	ACCACCTGACACGCTGATG	CCCCTGAGACTTAACCCTTTG
DDX21	CGTTCCGTAGGTGCCTTCTA	CTCTCGTCACTTCCCGTAGC
GAPDH	TACTAGCGGTTTTACGGGCG	TCGAACAGGAGGAGCAGAGAGCGA

Supplementary Table 2. Gene expression data after FLI1 silencing in GCB DLBCL cell lines.
(Worksheet A) Supervised analysis using limma of transcriptome and **(Worksheet B)** gene sets significantly enriched after FLI1 silencing in GCB DLBCL cell lines annotated with gProfiler.

Supplementary Table 3. FLI1 ChIP-Seq analysis in GCB DLBCL cell lines. (Worksheet A) Percentage of FLI1 peaks from GCB DLBCL cell lines and from literature. **(Worksheet B)** ChIP-Seq list of genes. **(Worksheet C)** List of unique genes from peaks within promoter.

Supplementary Table 4. Direct targets of FLI1. (Worksheet A) Genes identified after integration of RNA-Seq with ChIP-Seq data and **(Worksheet B)** their functional annotation obtained applying gProfiler.

Supplementary Table 5. Genes correlated with FLI1 in clinical samples and essential direct targets of FLI1 obtained after integration of RNA-Seq with ChIP-Seq. (Worksheet A) Genes correlated with FLI1 in clinical samples **(Worksheet B)** essential direct target genes of FLI1.

Supplementary Table 6. C-HiC annotation of FLI1 peaks. (Worksheet A1 and A2) VAL and OCI-Ly1 FLI1 peaks annotated with C-HiC. **(Worksheet B)** List of unique genes. **(Worksheet C)** Chromatin loops focus on ASB2.

Supplementary Table 7. Genes obtained after integration of RNA-Seq with C-HiC annotated FLI1 peaks. (Worksheet A) Indirect targets of FLI1, **(Worksheet B)** genes correlated with FLI1 in clinical samples and **(Worksheet C)** essential genes.

References

1. Reddy A, Zhang J, Davis NS, Moffitt AB, Love CL, Waldrop A, Leppa S, Pasanen A, Meriranta L, Karjalainen-Lindsberg ML, et al: **Genetic and Functional Drivers of Diffuse Large B Cell Lymphoma.** *Cell* 2017, **171:**481-494.e415.
